# miR-1249-5p regulates the osteogenic differentiation of ADSCs by targeting PDX1

**DOI:** 10.1186/s13018-020-02147-x

**Published:** 2021-01-06

**Authors:** Xiao-Mei Yang, Ya-Qi Song, Liang Li, Dong-Ming Liu, Guang-Dong Chen

**Affiliations:** 1 The Department of Emergency, Cangzhou Central Hospital, Cangzhou, 061000 China; 2 The Department of Orthopedics, Cangzhou Central Hospital, No. 16 Xinhua West Road, Cangzhou, 061000 Hebei Province China

**Keywords:** miR-1249-5p, Adipose derived stem cells, Osteogenic Differentiation, PDX1

## Abstract

**Background:**

Osteoporosis (OP) is an age-related systemic bone disease. MicroRNAs (miRNAs) are involved in the regulation of osteogenic differentiation. The purpose of this study was to explore the role and mechanism of miR-1249-5p for promoting osteogenic differentiation of adipose-derived stem cells (ADSCs).

**Methods:**

GSE74209 dataset was retrieved from NCBI Gene Expression Omnibus (GEO) database and performed bioinformatic analyses. OP tissue and healthy control tissues were obtained and used for RT-PCR analyses. ADSCs were incubated with miR-1249-5p mimic, inhibitor and corresponding negative control (NC), alkaline phosphatase (ALP) staining, and Alizarin Red Staining (ARS) were then performed to assess the role of miR-1249-5p for osteogenesis of ADSCs. Targetscan online website and dual-luciferase reporter assay were performed to verify that the 3′-UTR of PDX1 mRNA is a direct target of miR-1249-5p. RT-PCR and western blot were also performed to identify the mechanism of miR-1249-5p for osteogenesis of ADSCs.

**Results:**

A total of 170 differentially expressed miRNAs were selected, among which, 75 miRNAs were downregulated and 95 miRNAs were upregulated. Moreover, miR-1249-5p was decreased in OP patients, while showed a gradual increase with the extension of induction time. miR-1249-5p mimic significantly increased osteogenic differentiation capacity and p-PI3K and p-Akt protein levels. Luciferase activity in ADSCs co-transfected of miR-1249-5p mimic with PDX1-WT reporter plasmids was remarkably decreased, but there was no obvious change in miR-1249-5p mimic with PDX1-MUT reporter plasmids co-transfection group. Overexpression PDX1 could partially reverse the promotion effects of miR-1249-5p on osteogenesis of ADSCs.

**Conclusion:**

In conclusion, miR-1249-5p promotes osteogenic differentiation of ADSCs by targeting PDX1 through the PI3K/Akt signaling pathway.

## Background

Osteoporosis (OP) is an age-related systemic bone disease characterized by reduced bone mass and, damaged bone microstructure, which eventually leads to reduced bone strength and prone to fracture [1, 2]. In addition to a significant decrease in bone mass, high levels of bone marrow adipose tissue were also observed in patients with annual OP [3]. Therefore, senile osteoporosis is characterized by reduced bone mass in bone tissue and accumulation of fat tissue [4].

ADSCs can play the function of multidirectional differentiation and self-renewal, and can differentiate into osteoblasts, chondrocytes, adipocytes, nerve cells, and myoblasts under different conditions [5, 6]. In addition, ADSCs also have the advantages of easy access, simple sampling, minimally invasive, abundant sources, and relatively small immune rejection [7]. However, ADSCs have a low osteogenic efficiency and a great adipogenic tendency [8]. One possible method to promote osteogenic differentiation is to use of microRNAs (miRNAs) to promote osteogenic differentiation.

MiRNAs are a class of non-coding small RNA molecules that have been shown to function by negatively regulating target gene expression at transcriptional or post-transcriptional levels [9]. More and more evidence shown that miRNAs can be involved in regulating various molecular biological processes, such as cell proliferation [10], differentiation [11], tissue and organ development [12], and tumorigenesis [13].

Recently, several miRNAs have been found to play an important role in the balanced regulation of osteogenic and adipogenic differentiation. Zha et al. [14] found that miR-920 promotes osteogenic differentiation of bone mesenchymal stem cells by targeting HOXA7. Feng et al. [15] found that miR-378 suppressed osteogenesis of bone mesenchymal stem cells via interacting Wnt/β-catenin signaling pathway. However, little is known about the role of miRNAs for osteogenic of ADSCs.

In the present study, we downloaded and analyzed miRNA array data in the Gene Expression Omnibus (GEO) database (https://www.ncbi.nlm.nih.gov/geo). We found that miR-1249-5p was differentially expressed between OP and healthy controls. We then performed a series studies to explore the function of miR-1249-5p and its target gene.

## Material and methods

### Differentially expressed miRNAs in GSE74209

The miRNA array data of 6 postmenopausal women with osteoporosis and 6 healthy postmenopausal women in the GSE74209 dataset was retrieved from NCBI Gene Expression Omnibus (GEO) database. Quantile normalization and subsequent data processing were performed using the R software package. Differential expression analysis between OP and healthy control was performed using the R package limma. *P* < 0.05 and |fold change (FC)|≥ 1 was considered as statistically significant. A heatmap was constructed from the miRNA expression data using the pheatmap R package. FunRich software is used to carry out gene enrichment analysis for differentially miRNA-targeted genes with *P* < 0.01.

### OP tissue and healthy controls bone tissues

OP bone tissue was acquired from OP patients that prepared for surgery. Diagnosed criteria was in accordance with the World Health Organizations diagnostic criteria for osteoporosis, in which a patients *T*-score is less than or equal to − 2.5 (*T*-score ≤ − 2.5) [16]. Normal bone tissue was obtained from comminuted fractures patients that without OP. This study was obtained from ethics approval by Cangzhou Central Hospital and all patients gave consent to participate into this study.

### ADSCs isolation and osteogenic differentiation

Human ADSCs were isolated and cultured as previously described. In brief, adipose tissue was obtained from infrapatellar fat pad. Surrounding fascia and vessels were cut off and cut into 1 × 1 × 1 mm^3^ pieces with scissors. Then, these tissues were digested by 0.25% collagenase type II at 37 °C for 2 h. After filtration by aseptic cell sieves, the cells were washed and collected by centrifugation (500×*g*, 5 min, 3×) with Hank’s. Cells fewer than 5 generations old were used for experiments.

The ADSCs in 6 wells plate were cultured in osteogenic differentiation medium consisted of 10% FBS, 1 × 10^−8^ M dexamethasone, 10 mM β-glycerophosphate, and 50 μg/ml l-ascorbic acid.

### ADSCs transfection

ADSCs were seeded into 6-well plate (Corning, NY, USA) for 24 h prior to transfection. miR-1249-5p mimic and miR-1249-5p inhibitor were purchased from Ribobio (Guangzhou, China), as well as the negative controls. The full-length human PDX1 coding region was cloned into pcDNA3.1 (Invitrogen). Cell transfection of plasmids (3 μg) and oligonucleotides (100 nM) into RASFs was performed by Lipofectamine 3000 reagent (Invitrogen) according to the manufacturer’s instructions. In rescue experiments, co-transfection was launched with 1.5 μg plasmid and 60 nM miR-1249-5p mimic. Cells were subsequently cultured for another 30 h prior to further study.

### ALP and ARS

The ADSCs were divided into following groups: mimic NC, miR-1249-5p mimic, inhibitor NC, and miR-1249-5p inhibitor. All these four groups were cultured in osteogenic differentiation medium for 7 days. ALP activity was assessed using an ALP assay kit (Beyotime, Shanghai, China).

ALP staining was performed using BCIP/NBT Alkaline Phosphatase Color Development Kit (Beyotime Biotech Inc., Shanghai, China). In brief, ADSCs were fixed by 4% paraformaldehyde and then incubated with BCIP/NBT working solution for 30 min. Stained cells were observed under microscope and were photographed.

Alizarin Red S (ARS) staining (Solarbio, Beijing, China) was performed to assess the calcium deposition. In brief, ADSCs were induced for 21 days and fixed with 4% paraformaldehyde for 15 min. After washing with PBS for 3× and then stained with 1% Alizarin Red S (Solarbio, Beijing, China) solution for 30 min. Stained cells were observed under Olympus microscope (Tokyo, Japan) and were photographed.

### Quantitative real-time PCR (qRT-PCR)

Total RNA from ADSCs were extracted with Qiagen miRNeasy Mini kit (Qiagen, Hilden, Germany) following the manufacturer’s protocol. Then, we reverse transcribed RNA to cDNA (Takara, Dalian, China) following the manufacturer’s instruction. Quantitative RT-PCR was then performed by One-Step SYBR PrimeScript RT PCR Kit II (Takara, Dalian, China). The relative expression levels of miR-1249-5p and PDX1 mRNA were calculated by 2^−ΔΔCT^ methods with normalization to U6 small nuclear RNA (U6) and glyceraldehyde-3-phosphate dehydrogenase (GAPDH), respectively. All PCR reactions were performed in triplicate. Primers were as shown in Table 1.

**Table 1:** Primer sequences for qRT-PCR

Gene	Forward primer 5′-3′	Reverse primer 5′-3′
GAPDH	GACAGTCAGCCGCATCTTCT	GCGCCCAATACGACCAAATC
U6	GGCTTTTTGCGGTCTGG	GCTTCGGCAGCACATATACTAAAAT
miR-1249-5p	ACACTCCAGCTGGGTTCACAGTGGCTAAG	AGGGCTTAGCTGCTTGTGAGCA
OSX	AGACCTCCAGAGAGGAGAGAC	GGGGACTGGAGCCATAGTGA
ALP	AATAGCCCTGGCAGATTCCC	CTCTCATGGTGTCTCGGTGG
PDX1	TGCCACTGTTGAGTGCAAGTC	ACCTGGAGAAGCCGAAGGTAA

### Western blot

The intracellular protein was collected by digestion and centrifugation, washed twice with PBS, and the cell pellet was collected by centrifugation. A certain amount of lysate was added and lysed on ice for 30 min. After lysing, the total protein was collected by centrifugation at 12,000 r/min for 15 min at 4 °C. A certain amount of protein loading buffer was added, and the mixture was denatured at 100 °C for 15 min, and then polyacrylamide gel electrophoresis (12%, SDS-PAGE) was performed. After electrophoresis, the proteins were transferred to a 4.5-μm polyvinylidene fluoride (PVDF) membrane. The PVDF membrane was blocked with 5% non-fat milk for 1 h at room temperature, and a primary antibody ALP (Abcam, 1:1000), OSX (Abcam, 1:1000), AKT (Abcam, 1:1000), p-AKT (Abcam, 1:1000), PI3K (Abcam, 1:1000), p-PI3K (Abcam, 1:500), and GAPDH (Abcam, 1:2000) were added to incubate overnight. After incubation, the PVDF membrane was washed 3× with TBST buffer (TRIS-HCl balanced salt buffer + Tween), and then incubated with the secondary antibody (IgG H&L (HRP), Abcam, 1:2000) at room temperature in the dark for 1 h. The bands were then washed three times with TBST buffer, the membrane was then scanned with an Odyssey VI scanner (Li-Cor Biosciences), and the molecular weight and optical density values of the target bands were analyzed using a gel image processing system.

### Luciferase assay

PDX1 3′UTR containing binding sites of miR-1249-5p was cloned by PCR methods into psi-CHECK vector (Invitrogen), as well as the mutated sequences (named PDX1 3′UTR-WT/MUT). ADSCs were co-transfected with PDX1 3′UTR-WT/MUT and miR-1249-5p/NC mimic or miR-1249-5p/NC inhibitor. After 48 h incubation, ADSCs were collected to measure Firefly and Renilla luciferase activity using the dual-luciferase reporter assay system (Promega, Madison, WI, USA). All the data were the average of at least three independent transfections and presented as fold change normalized to control group (3′UTR-WT+miR-NC mimic/inhibitor).

### Statistic analysis

Student’s *t* test or one-way analysis of variance (ANOVA) of SPSS statistical software (version 2.0; SPSS, Inc., Chicago, IL, USA) was used for statistical analysis, and the results were expressed by mean ± standard deviation. The difference was statistically significant at *P* < 0.05.

## Results

### Bioinformatic analysis to identify differentially expressed miRNAs

**Fig. 1:**
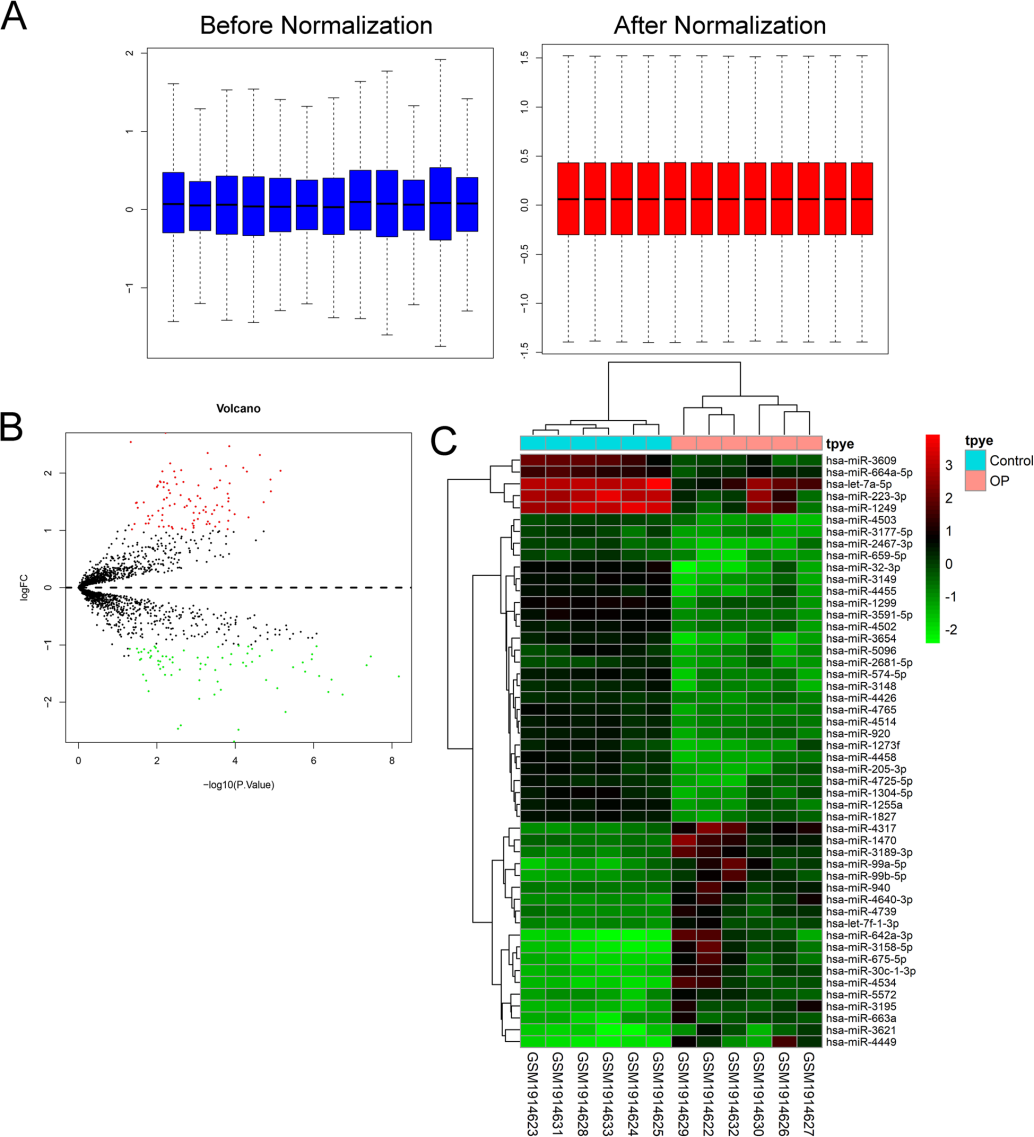
Bioinformatic analysis of GSE74209. **a** Comparison of expression value between before normalization and after normalization. **b** The volcano plot screen differentially expressed miRNAs between OP and healthy controls. **c** Heatmap of the top 50 differentially expressed miRNAs between OP patients and healthy control

**Fig. 2:**
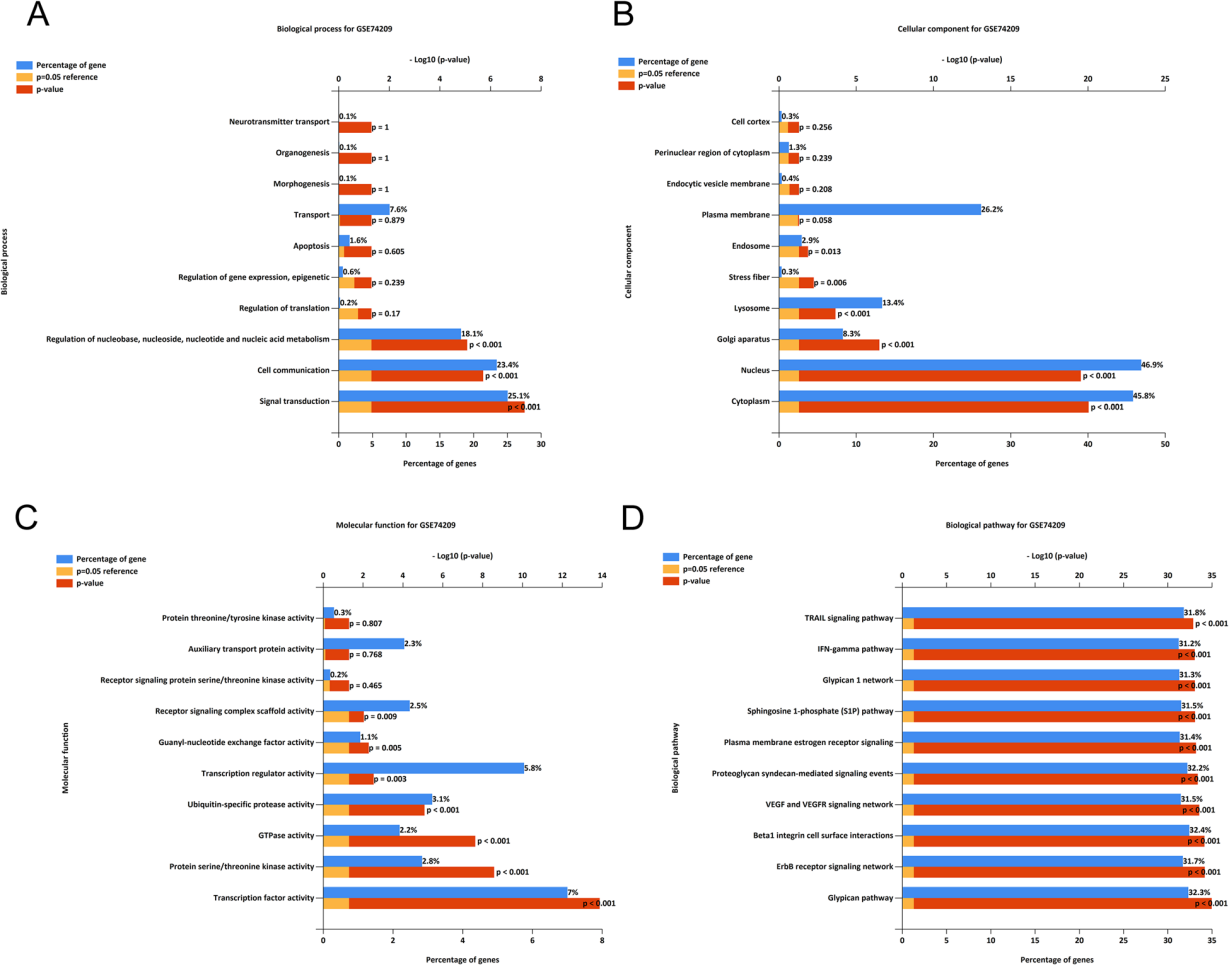
Gene function of miR-1249-5p enriched by Funrich software. **a** Biological process of the target genes of miR-1249-5p. **b** Cellular component of the target genes of miR-1249-5p. **c** Molecular function of the target genes of miR-1249-5p. **d** KEGG pathway of the target genes of miR-1249-5p

A box plot of the data before and after normalization is shown in Fig. 1a. The median values of each sample were extremely similar, indicating that the data should be further analyzed. A total of 170 differentially expressed miRNAs between OP patients and healthy controls, among which, 75 miRNAs were downregulated and 95 miRNAs were upregulated. The volcano plot and heatmap of differentially expressed miRNAs are shown in Fig. 1b, c, respectively. MiR-1249-5p was the most statistically significant downregulated miRNAs in OP patients. The miRNA enrichment analysis was determined via Funrich software according to biological process. GO biological process (BP) terms were mainly enriched in neurotransmitter transport, organogenesis, morphogenesis, transport, apoptosis, regulation of gene expression, epigenetic, regulation of translation, regulation of nucleobase, nucleoside, nucleoside and nucleic acid metabolism, cell communication, and signal transduction (Fig. 2a). GO cellular component (CC) terms were mainly enriched in cell cortex, perinuclear region of cytoplasm, endocytic vesicle membrane, plasma membrane, endosome, stress fiber, lysosome, golgi apparatus, nucleus, and cytoplasm (Fig. 2b). GO molecular function (MF) terms were mainly enriched in protein threonine/tyrosine kinase activity, auxiliary transport protein activity, receptor signaling protein serine/threonine kinase activity, receptor signaling complex scaffold activity, guanyl-nucleotide exchange factor activity, transcription regulator activity, ubiquitin-specific protease activity, and GTPase activity (Fig. 2c). KEGG terms were mainly enriched in TRAIL signaling pathway, IFN-gamma pathway, glypican 1 network, sphingosine 1-phosphate (S1P) pathway, plasma membrane estrogen receptor signaling, proteoglycan syndecan-mediated signaling events, VEGF and VEGFR signaling network, Beta1 integrin cell surface interactions, ErbB receptor signaling network, and glypican pathway (Fig. 2d).

### miR-1249-5p was downregulated in OP patients

**Fig. 3:**
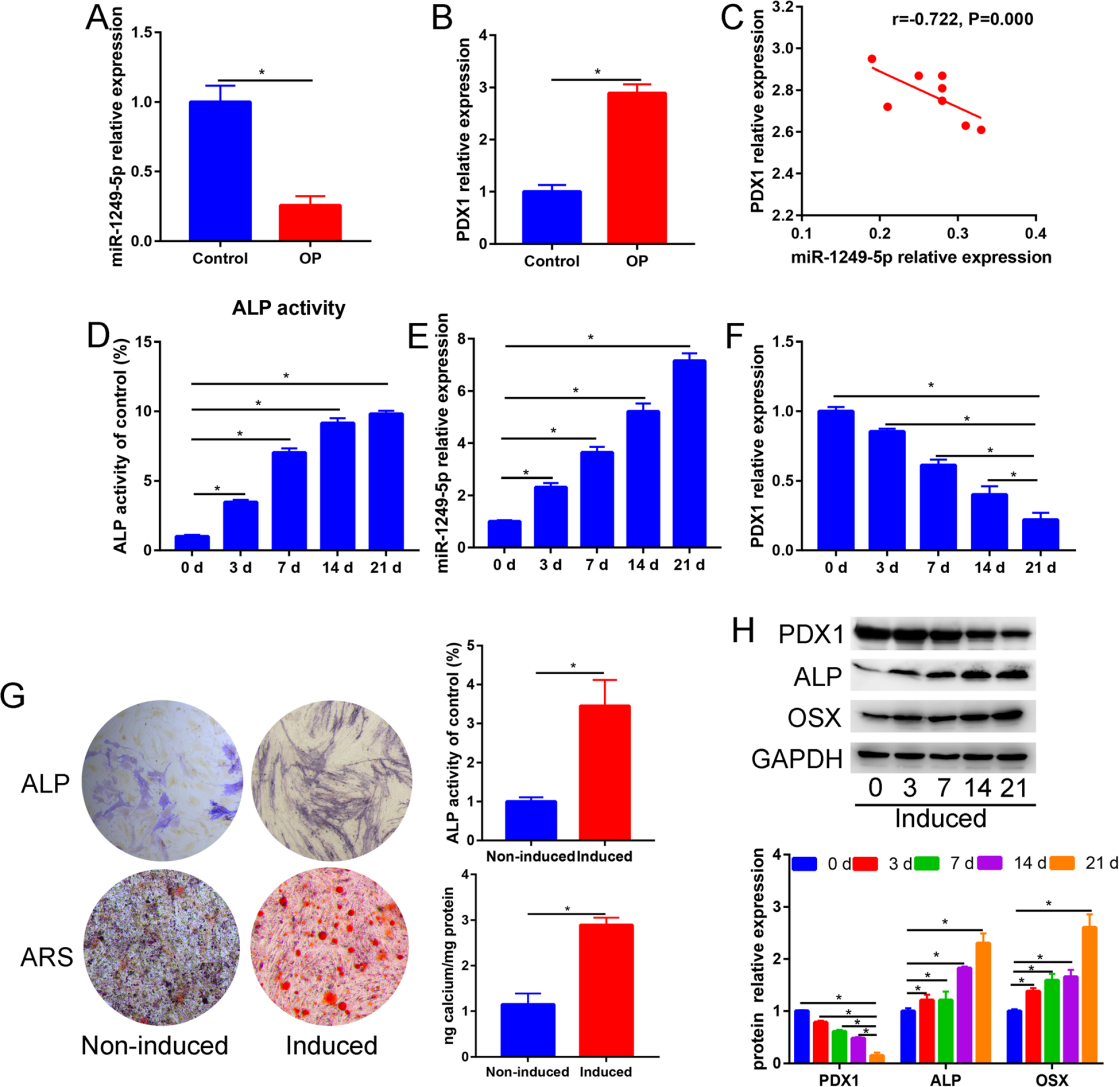
miR-1249-5p was downregulated in OP patients. **a** Relative expression of miR-1249-5p in OP patients and healthy controls. **b** Relative expression of PDX1 in OP patients and healthy controls. **c** Correlation analysis of miR-1249-5p and PDX1 in OP patients. Relative ALP activity (**d**), miR-1249-5p (**e**), and PDX1 (**f**) during the osteogenic differentiation of ADSCs at 0, 3, 7, 14, and 21 days. **g** Alkaline phosphatase (ALP) and Alizarin Red staining (ARS) between non-induced and induced ADSCs. **h** Protein expression of PDX1, ALP, and OSX during the osteogenic differentiation of ADSCs at 0, 3, 7, 14, and 21 days. *P < 0.05

To verify the results of the miRNAs microarray, we performed a real-time quantitative PCR assay. Result found that miR-1249-5p was significantly downregulated in OP patients than that of healthy controls (*P* < 0.05, Fig. 3a). In contrast, the relative expression of PDX1 was significantly upregulated in OP patients than that of healthy controls (*P* < 0.05, Fig. 3b). Pearson correlation analysis found a significant negative correlation (*r* = − 0.722, *P* = 0.000, Fig. 3c) between miR-1249-5p and PDX1 in OP patients. ALP activity and miR-1249-5p expression of the ADSCs group showed a gradual increase with the extension of induction time (Fig. 3d, e, respectively), and got close to the extreme at day 21. While PDX1 expression of the ADSCs group showed a gradual decrease with the extension of induction time (Fig. 3f). Subsequently, we performed ALP and ARS staining assays after culture with osteogenic-induced media. ADSCs in induced group had higher ALP activity and calcium deposition than the control group (Fig. 3g). As the induction time progressed, the protein expression of ALP and OSX gradually increased and the protein expression of PDX1 gradually decreased (Fig. 3h).

### miR-1249-5p promoted ADSCs osteogenic differentiation of ADSCs

**Fig. 4:**
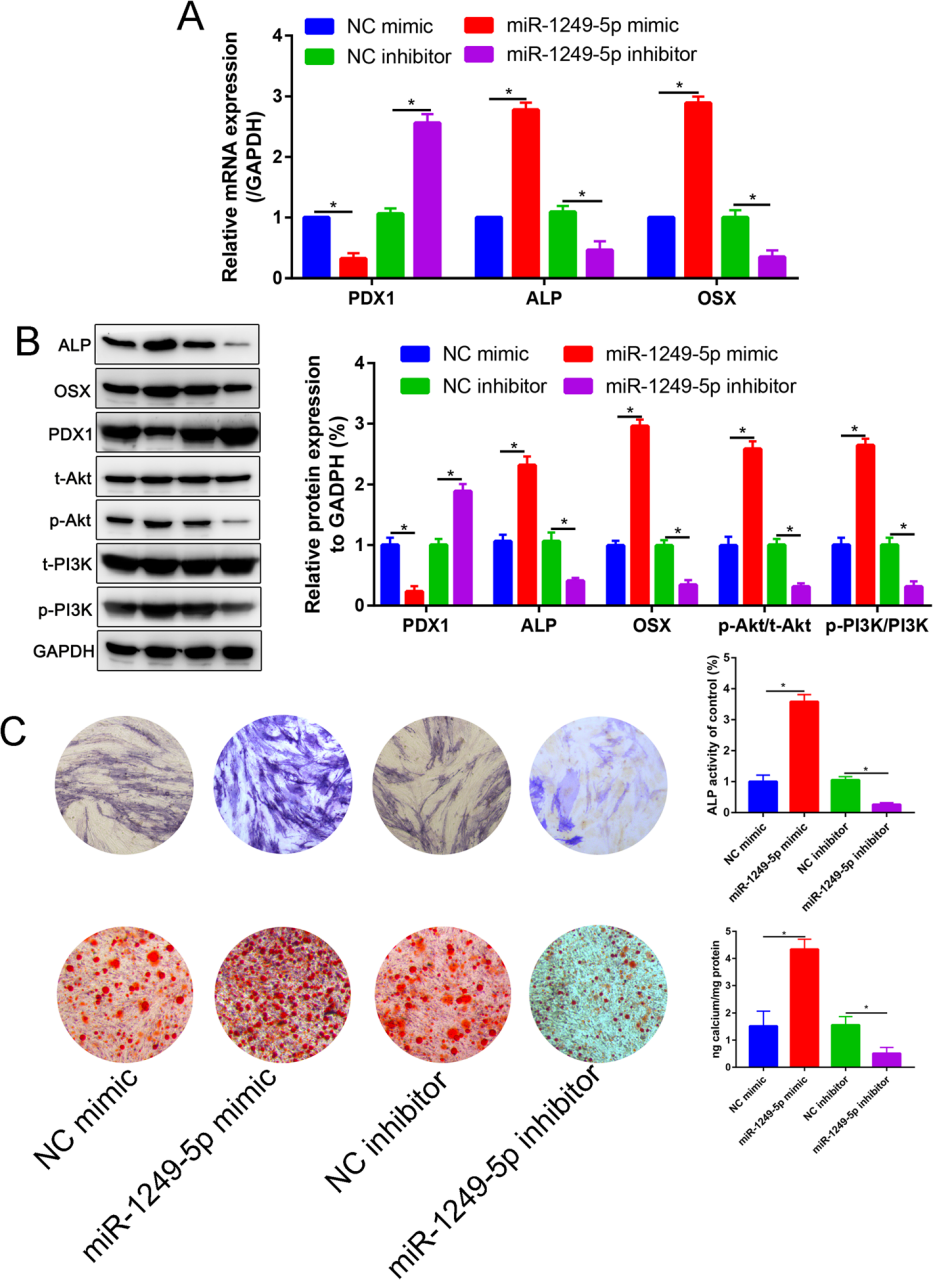
miR-1249-5p enhance the osteogenic differentiation of ADSCs. **a** PDX1, ALP, and OSX mRNA expression were detected by real-time RT-PCR. **b** PDX1, ALP, OSX, t-Akt, p-Akt, PI3K, and p-PI3K protein expressions were measured by western blot assay. **c** Alkaline phosphatase (ALP) and Alizarin Red staining (ARS) of miR-1249-5p mimic, inhibitor, and corresponding negative control after 7 days and 21 days differentiation respectively. *P < 0.05

qRT-PCR data showed that the gene expression levels of ALP and OSX increased following transfection of miR-1249-5p mimic compared with transfection of NC mimic, whereas they decreased markedly following transfection with miR-1249-5p inhibitor compared with the transfection of inhibitor NC (Fig. 4a). qRT-PCR data showed that the gene expression levels of PDX1 increased following transfection of miR-1249-5p mimic compared with transfection of NC mimic, whereas decreased markedly following transfection with miR-1249-5p inhibitor compared with the transfection of inhibitor NC (Fig. 4a). The western blotting results were consistent with the qRT-PCR results (Fig. 4b). Moreover, western blot analysis was conducted to compare the protein levels of p-PI3K, PI3K, p-Akt, and Akt in the ADSCs that transfected with miR-1249-5p mimics, inhibitor, and corresponding NC. The data indicated that the expression levels of p-PI3K and p-Akt, in ADSCs transfected with miR-1249-5p mimics group, were upregulated compared with those in the NC mimic group (*P* < 0.05), whereas they decreased markedly following transfection with miR-1249-5p inhibitor compared with the transfection of inhibitor NC (*P* < 0.05). ALP staining and ARS staining were used to investigate the ability of osteogenesis in ADSCs transfected with miR-1249-5p mimic or inhibitor. ALP activity and calcium deposition was significantly increased in miR-1249-5p mimic and decreased in miR-1249-5p inhibitor than corresponding NC. These results suggested that miR-1249-5p promotes osteogenic differentiation of ADSCs through PI3K/Akt signaling pathway.

### PDX1 was a direct target of miR-1249-5p

**Fig. 5:**
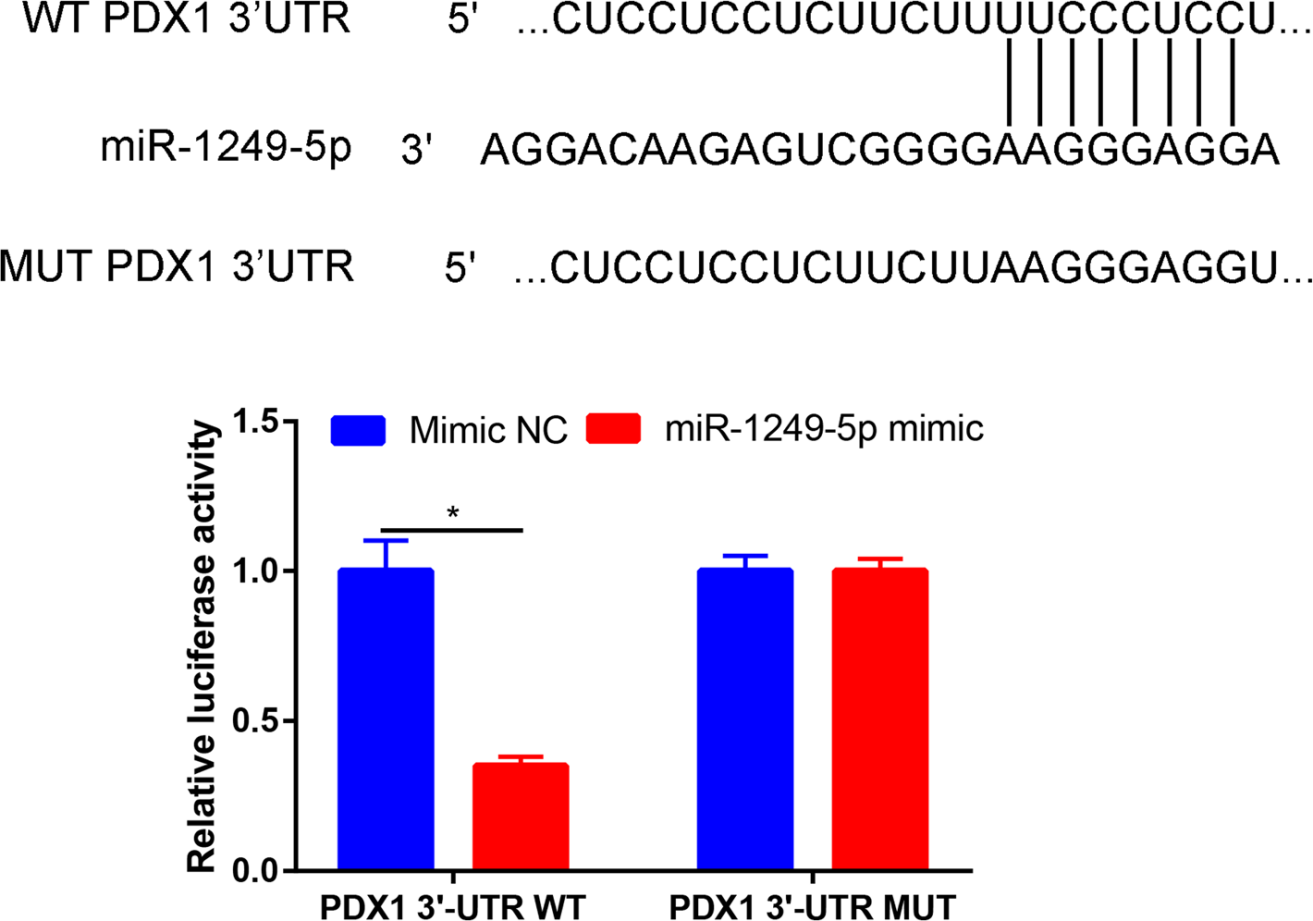
miR-1249-5p directly target with PDX1. The binding sites of miR-1249-5p with the 3′UTR of PDX1 and dual-luciferase activity in the wild type and mutant PDX1 3′UTRs in 293T cells treated with miR-1249-5p mimic and control. *P < 0.05

Based on TargetScan 7.1, we identified that PDX1 was a potential target of miR-1249-5p (Fig. 5a). Then, we used the luciferase reporter assay to verify whether PDX1 directly targeted miR-1249-5p. We observed that the luciferase activity in ADSCs co-transfected of miR-1249-5p mimic with PDX1-WT reporter plasmids was remarkably decreased, but there was no obvious change in miR-1249-5p mimic with PDX1-MUT reporter plasmids co-transfection group (Fig.5b).

### Overexpression of PDX1 partially reverse the osteogenic promotion effects of miR-1249-5p

**Fig. 6:**
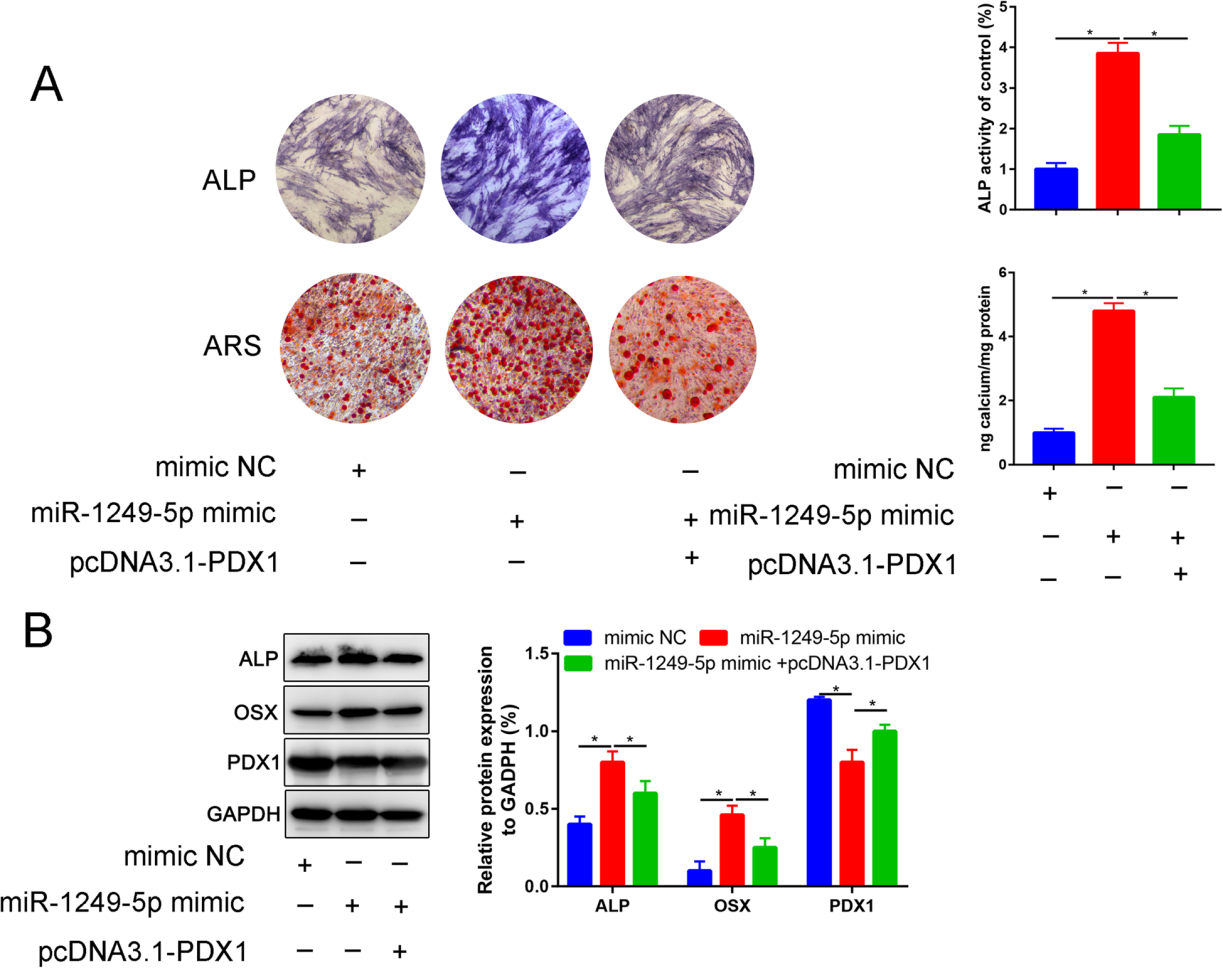
Overexpression PDX1 partially reverse the promotion effect of miR-1249-5p for osteogenesis of ADSCs. **a** Alkaline phosphatase (ALP) and Alizarin Red staining (ARS) of mimic NC, miR-1249-5p mimic, and miR-1249-5p mimic combined with pcDNA3.1-PDX1 after 7 and 21 days differentiation, respectively. **b** Relative protein expression of ALP, OSX, and PDX1 in mimic NC, miR-1249-5p mimic, and miR-1249-5p mimic combined with pcDNA3.1-PDX1 groups. *P < 0.05

miR-1249-5p mimic upregulated ALP activity and mineralization ability in ADSCs undergoing osteogenic differentiation, which were partially reversed by overexpression of PDX1 (Fig. 6a). miR-1249-5p mimic upregulated ALP and OSX and attenuated PDX1 expression in ADSCs undergoing osteogenesis, which were partially reversed by overexpression of PDX1 (Fig. 6b).

## Discussion

The pathogenesis of OP is caused by the imbalance between osteoblasts and osteoclasts. When osteoclasts are more active than osteoblasts, it will lead to the decrease of bone density, which in turn will lead to osteoporosis. ADSCs have the ability to differentiate into multiple cells under specific induction conditions in vitro.

In recent years, a large number of studies have shown that ADSCs can be differentiated into osteoblasts under certain environmental induction. Due to their characteristics of easy extraction and isolation and large proliferation in vitro, ADSCs have become a kind of seed cells that are expected to participate in the clinical treatment of osteoporosis. Improving the osteogenic differentiation ability of ADSCs is the key to bone regeneration.

Studies have confirmed that several miRNAs can influence the osteogenic differentiation of ADSCs, such as miR-145-5p [17], miR-143-3p [18], and miR-129-5p [5]. Wang et al. [19] reported that overexpression of miR-346 could regulate the Wnt/β-catenin signaling pathway by targeting GSK3b to promote the differentiation of BMSCs into osteoblasts.

It was also found that the expression level of miR-20a increased gradually during the osteogenic differentiation of BMSCs into osteoblasts. Further research found that miR-20a directly target with PPARγ and finally regulate BMP/Runx2 signaling pathway to promote the osteogenic differentiation of BMSCs [20].

The expression level of miR-1249-5p was significantly downregulated in patients with OP in GEO dataset. The results suggest that increased expression of miR-1249-5p may improve the osteogenic differentiation of ADSCs. miR-1249-5p is a miRNA molecule that is widely expressed and highly conserved in human, horse, mouse, and other animal tissues. miR-1249-5p plays an important regulatory role in inflammation [21], tumor progression [22], and cell differentiation [23]. In this study, we firstly identified that miR-1249-5p was downregulated in OP patients than that of healthy controls. This reminds us that miR-1249-5p may be a promising target for OP. We therefore explore the promotion effects of miR-1249-5p on osteogenesis of ADSCs. ALP staining and ARS found that miR-1249-5p could enhance the ALP activity and calcium deposition. These results suggested that miR-1249-5p has a positive role in promoting the osteogenic differentiation of ADSCs. It has been demonstrated that miRNA functions by inhibiting the expression of targeted genes [24]. Dual-luciferase reporter assays showed that miR-1249-5p binds directly to the PDX1 3′UTR. PDX1 is known to play an important role in cell development and cell differentiation [25]. We firstly identify the function and mechanism of PDX1 in osteogenic differentiation of ADSCs. PDX1 was gradually decreased with the increase of the induction time. Moreover, overexpression PDX1 could partially reverse the effects of miR-1249-5p on osteogenic differentiation of ADSCs. The main limitation of the present study was the lack of in vivo experiments to verify the role of miR-1249-5p for regulating osteogenesis.

## Conclusion

In conclusion, this study firstly demonstrated that miR-1249-5p could enhance the osteogenesis of ADSCs in vitro through PI3K/Akt signaling pathway. PDX1 was identified as novel target of miR-1249-5p. Our study provided a new insight into the treatment of OP.

## Data Availability

We state that the data will not be shared since all the raw data are present in the figures included in the article.

## Abbreviations

OP: Osteoporosis
miRNAs: MicroRNAs
ADSCs: Adipose-derived stem cells
GEO: Gene Expression Omnibus
NC: Negative control
ALP: Alkaline phosphatase
ARS: Alizarin Red Staining
FC: Fold change
BCA: Bicinchoninic acid
qRT-PCR: Quantitative real-time PCR
GAPDH: Glyceraldehyde-3-phosphate dehydrogenase
PVDF: Polyvinylidene fluoride
ANOVA: One-way analysis of variance
BP: Biological process
CC: Cellular component
MF: Molecular function
